# Address

**Published:** 1876-04

**Authors:** W. W. H. Thackston


### AMERICAN JOURNAL

OF

### DENTAL SCIENCE.

Vol. IX. THIRD SERIES—APRIL, 1876. No. 12.

### ARTICLE I.

### *Address*—Concluded.

BY W. W. H. THACKSTON, M. D., D. D. S.

Delivered before the Virginia State Dental Association.

It is nothing to the discredit of modern medicine that its formerrepresentatives were quacks and charlatans, barbers, cuppers and leechers; and it is nothing to the disgrace or reproach of modern dentistry that *its* practitioners of the olden time were strollers, mechanics, tradesmen and tricksters. Both are now honorable and indispensable professions; while both must acknowledge a very obscure and humble origin; and for a time, a very discreditable history and following.

We admit the fact that dental surgery as a profession is but yet in its youth—in the "green tree," while medicine is comparatively hoary with age, and teeming with honors; we concede our dependence upon several of the departments of medical science for the correct apprehension and successful practice of dental surgery, just as the portrait painter and sculptor are dependent upon anatomy, for the proper treatment of *their* subjects—as the pharmacist is dependent upon chemistry and Materia Medica, and the criminal lawyer upon toxicology and medical jurisprudence, as a preparation for their several pursuits. And while we do not depreciate or undervalue the departments of medicine forming an integral part of our professional training, yet *of* themselves and *by* themselves, they can no more make, or qualify the *dentist*, than will the possession of a medical degree qualify or evidence the painter, the sculptor, the pharmacist or the lawyer.

While, in many senses, the relations of medicine and dental surgery are close and intimate, there are nevertheless broad and ineffaceable lines of distinction, which individualize and will forever separate the two; and these differences are inherent, educational and practical. The M. D. may become a safe and skilful dentist, but *not* because he is a thoroughly qualified physician: the "D. D. S.," may become a respectable general surgeon, or practitioner of medicine, but *not* because of his attainments or skill as a dentist. All this we assert and admit with cheerful frankness and unreserve, but we do not admit that we are dependent as individuals, or as a profession, upon any other class of men for our social or professional status. We do not admit that it is politic, or wise, or dignified, to be clamoring for admission into outside organizations, or compatible with a becoming regard for our calling, or respect for ourselves, to be dancing attendance upon medical men for the poor boon of professional recognition.

We beg to suggest to our restless and dissatisfied brethren, that the readiest, the most certain, as well as the more manly and honorable means of securing the consideration for which they sigh and groan, will be to *deserve* recognition by the display of superior talents and attainments *as dentists*, and by the exhibition of virtuous and stainless lives and characters as men. These will command and secure all the respect and consideration an enlightened and elevated character can covet or desire. And we beg further to suggest, that the "flunkeyism," the self depreciation, and medical boot-licking which has distinguished this movement can legitimately secure no other result than the contempt, the derision and the disgust, not only of *true* medical men, but of intelligent and elevated society everywhere.

We trust, gentlemen, that if you have entertained any doubts upon the subject, we have satisfied you that dental surgery *is now* a profession; and that our distinctive college degree *is* a valid, expressive and appropriate degree; that it represents and expresses in dental surgery *all* that "M. D." does in medicine; and a great deal more even in medicine, than the "M. D." can possibly do in dental surgery; for the educated dentist *does know* something of medicine, while the physician is untaught, and absolutely ignorant of the very rudiments of dental surgery.

Let the purely "M. D." dentist enter the arena of practice, and however skilful he may diagnose disease, or write prescriptions, or saw bones, he will find himself unable to save or replace a human tooth; he may successfully reduce dislocations, and adjust fractures; but the simplest operation in dental surgery will overtax his ability and exhaust his resources; and U. F.—"Utter Failure"—will be the appropriate badge, or symbol of *his* very partial and most inadequate culture for the duties and offices of the dentist.

And now, gentlemen, permit me to say a few words about our educational system, and the reckless stupidity and criminal folly of any attempt to disrupt, or break-down, or radically change that system, for any of the old and tried and exploded systems of the past; or for any doubtful or questionable plan or method or experiment of the present.

It is proposed in some quarters, not only to abolish our college degree, but to abolish the colleges, and dissolve our faculties of instruction,—to go back, and again attach ourselves as a caudal appendage to the purely medical institutions of the country—thus yielding the vantage ground, and surrendering the prestige we now enjoy, and for which we have paid the sacrifice of labor, of money, and the lives of our best men of the nineteenth century.

Now, gentlemen, to be brief and concise, from the days of *Fauchard* to the establishment of the Baltimore College, a period of over a hundred years, the medical schools were open all over the world to students of dental surgery; many availed themselves of all the facilities and resources those schools afforded; and what was the result? What sort of dentists did they make? And what was the status and progress of dental surgery under that regime? You may count upon your fingers every dentist who in that lapse of time, achieved individual distinction, or in any sense, advanced dental science. "A baker's dozen" will comprise every name that history honors with a passing notice. Dental surgery was then indeed and in truth, with few and rare exceptions, a business, a trade, and an occult art. Character it had, but it was the character of the empiric and charlatan, of the impostor and trickster. But in 1839-40 a new departure was taken, a college of dental surgery was incorporated and put in operation, the American Association of Dental Surgeons was organized; with Harris, and Hayden and Parmly at its head; the American Journal of Dental Science was established and published; and what has been the effect on dental science; not only in this country, but all over the civilized world? The results are before you, and all around you.

I shall not consume your time by describing the progress and improvements in dental science during the past three decades, and I need not pause to compare the present social status of the dentist, with the period when the very *epithet* carried to its miserable wearer the distrust and suspicion of society. *Then*, our representives were only too happy to be acknowledged as respectable and trustworthy; now, they are so elevated, that with some of them, nothing short of recognition as physicians and general surgeons will satisfiy their vaulting ambition.

What warrant or assurance have we, that we shall better our condition or improve our prospects by striking down the institutions and influences that have made us what we are? That have given to the title of "American Dentist," a world wide celebrity and distinction, and returning to the old, the effete, and abandoned system, that choked and stifled all progress; that covered us with the rags and tatters of professional garniture, and held us for one hundred years under the ban of professional outlawry and social ostracism?

We confess, gentlemen, that we can find nothing to base even a rational hope upon; we can find nothing in the arguments in favor of exclusive medical training, and purely medical schools, and nothing in the ungenerous and disparaging assaults upon our special and distinctive system and schools, but the abiding conviction, that the movement is suicidal, and that its success would be the very "consummation of madness and folly."

Let us then stand by our present system and agencies as the palladium of our safety, the anchor of our hopes, and let us accept the startling and brilliant progress of the past, as earnest of the prospective and more brilliant achievements of the future.

We know that our colleges have been sharply criticised, and our faculties of instruction severely censured, perhaps in some instances deservedly; but often causelessly, *ignorantly*, and *unjustly*. We are not the apologist of the shortcomings and delinquencies of our schools; we know that the Alumni and graduates have not always reflected credit upon their *Alma Mater;* nay more, they have sometimes been a living and standing reproach to their professsion; but it should be remembered, that exceptionally, the material our professors have had to mold and work up, has not been of the very best; many of the matriculates of these schools are poorly prepared, and labor under disadvantages that no ability or fidelity on the part of the teacher can possibly compensate; defective primary education, lack of application and want of natural capacity, are the most common of the difficulties our college professors have to encounter. It is no easy task to force ideas through a thick skull, or provide brains for an empty head; that is a feat that not even medical schools have essayed, or at least successfully performed.

But in all these regards there is no appreciable difference between our own and all other schools. Other faculties are as often incompetent and delinquent as ours, and their graduates and degree men as variable in attainments, in character and in capacity as ours. It is no fair or just argument against a system, or a particular school, that we now and then encounter a graduate who is worthless and incompetent. Judged by such a test, *no* school in science or in letters could stand, and it is simply unreasonable and absurd, to expect or demand of our institutions, an infallibility never approached, or even contemplated in any department of human learning.

Our peculiar system of preparatory training was maturely considered, and wisely arranged by the distinguished and venerable men who created our profession. Every essential and available department of science was made tributary to the course of study adopted; subsequently, as improvement and advances have been made, the curriculum has been extended, and the corps of instructors strengthened. Beginning with a faculty of four professors, the course has broadened and lengthened, until now, the staff of instructors, professors, demonstrators and adjunct professors, number from ten to twelve in each school, and the most complete and liberal arrangements for infirmary practice and clinical instruction have been provided. We draw our medical instructors from the ranks of competent and qualified physicians; and it is simply puerile and foolish to say, that these men cannot, and do not teach their several departments as successfully and thoroughly in one school as in the other; and it is equally absurd and senseless to say that the dentist could lecture and instruct more satisfactorily and efficiently in a medical school, than in the halls of his own academy or college. And we ask again "*Cui Bono?*" What advantage, what compensating benefit can we reasonably anticipate from the proposed change? We confess, *we* can see none, we can rationally hope for none.

But we do see, that in our own schools, dental surgery as a unit, as a unified and complete science and profession, is the *leading* and distinguishing object and idea; while in the medical schools it would *not* be the leading object or idea; but a mere secondary, and subordinate appendage, confering no dignity or character, and exciting no special interest or concern with either teachers or students. We ask you then, gentlemen, as you value the present interest and future welfare of your chosen profession, as you value its importance to yourselves, and its worth to humanity; to sustain and uphold your *present* educational institutions; support them by your personal patronage and influence, and when called upon, give them a portion of your time, your labor and your money.

We admit, that some mistakes have been made in carrying out the work conceived and organized by Harris and Hayden, but these errors will in time work their own correction by the force of natural laws, which sooner or later, always assert their supremacy. Without reference to any special or particular institution, and in altogether a general sense, we must say, that we have at present, manifestly too many schools within. Certain limits, and under certain conditions, competition exercises a wholesome and vital influence; beyond those limits and conditions the maxim, that "competition is the life of trade," or the life of anything else, is a *fallacy*, and this fallacy has been most conclusively, and most sadly illustrated in the history and condition of our struggling and languishing colleges.

Beyond prescribed and well defined limits and conditions, competition is blight, and mill-dew, and death. The population of our country is too sparse—the demand for educated dentists is yet too limited, and the pecuniary resources of our people too scant to sustain the present number of our distinctive schools; and it is a sad and melancholy spectacle, to see good men frittering away their strength, and exhausting their energies upon classes so small, as to afford the instructors no compensation, save the consciousness of having faithfully performed a "labor of love." Carry out, or slightly extend this principle of competition; establish a school for dentists in every county of the State, make every tenth, or even twentieth man a dentist, and how long would the schools or dentists live?

But, as we already have these institutions in operation; if we cannot induce them to combine and consolidate, and thus bring the principle of competition within healthy limits, as was the desire of the Southern Association five years ago, why, we must do the best we can with, and for them, and let the future determine the destiny of each. We do not discriminate, or mean to be invidious. We have now in the South and South-west, four or five of these schools, all we believe, doing their best to deserve success, to command respect, and to perform thoroughly their appropriate work; and it should be our duty and pleasure, as it is manifestly our interest and advantage to sustain and encourage them by our sympathy and cordial co-operation.

And in this immediate connection we will say, that we cannot more effectively aid these institutions and enterprises, than by exercising care and discrimination in accepting students. We have told you on a former occasion "that relatively few men are capable of being made respectable or skillful dentists by any method or system of training; and when we are approached by one manifestly wanting in the essential qualities and attributes demanded by the needs of dental surgery, we should frankly and earnestly direct his attention to some other pursuit, suited to his talents and aptitudes."

While we should make a fair academic education an indispensable condition, and while we acknowledge the value and convenience of private office instruction, we honestly believe that our present college laboratories and infirmaries afford the very best means of preparation that a student can possibly avail himself, to profit by the regular lecture terms.

In a private office, if the dentist is well qualified to teach, his time is too valuable to himself, and too much occupied with the demands of his practice to be devoted, except to a very limited extent, to the instruction of a student; nor can he afford, or will he be permitted to turn over, or entrust his patients to the care of a novice.

By far the best course for all parties, and for every interest involved, will be to send our students to the college laboratories and infirmaries, where they can be prepared and qualified for the regular lecture terms under the guidance and supervision of the adjunct professors and demonstrators, with all the advantages of laboratory practice and experience; and the more important benefits of clinical observation and assistance. Our schools would thus secure additional support, our students incalculable advantages, and the private practitioners released from a burden and responsibility that few competent to teach, can afford to incur.

In conclusion, gentlemen, we are persuaded, that with our present resources and facilities, with the vantage ground we have gained and now occupy, with concentrated effort, and determined purpose to stand by our colors and to recruit our ranks with the better class of men that we are now attracting to our schools, dental surgery will maintain its status as a progressive, liberal and learned profession; and without any entangling alliance, or subordinate relation to any other profession, transmit its distinguishing characteristics, its honors and its benefactions, not only to our immediate successors, but to remotest posterity.

